# Transendothelial Transport of Low-Density Lipoproteins in Atherosclerosis: Mechanisms of Activation and Possible Therapeutic Targets

**DOI:** 10.3390/ijms27219492

**Published:** 2026-10-24

**Authors:** Dmitry Tanyanskiy, Alexander Denisenko

**Affiliations:** Department of Biological and Medical Chemistry, Institute of Experimental Medicine, 12, Acad. Pavlov Street, St. Petersburg 197022, Russia

**Keywords:** atherosclerosis, endothelial cells, low-density lipoproteins, LDL receptors, endothelial transcytosis

## Abstract

Despite advances in the prevention of cardiovascular events in recent decades, the risk of developing atherosclerosis and its complications remains a major challenge. One promising way to reduce this risk is to develop measures to inhibit the activated transport of low-density lipoproteins (LDLs) through the endothelium, which is the initial stage of atherosclerosis. Recent research has provided some molecular details of the mechanism, including the roles of caveolin-1, LDL receptors, and certain motor proteins. This review summarizes data on recent advances in studying of LDL endothelial transcytosis, focusing on its role in atherogenesis as well as on its regulation by hemodynamics, lipoproteins, cytokines, and metabolic factors. Data on potential targets for inhibiting transendothelial LDL transport are also presented. A better understanding of the mechanisms of endothelial LDL transcytosis and the development of drugs to inhibit it may lead to improved treatment of atherosclerosis and more effective prevention of cardiovascular events.

## 1. Introduction

Despite the significant progress made in the treatment of atherosclerosis over the past three to four decades, this disease and its clinical manifestations continue to be the leading cause of mortality worldwide [1]. According to forecasts, mortality from these conditions is expected to increase by 2050 [2]. For patients with clinical manifestations of atherosclerosis, treatment with statins and other medications, lifestyle changes to manage risk factors, and surgical procedures on the coronary and carotid arteries improve their life expectancy. However, despite these efforts, many patients still face the risk of atherosclerosis progression. To prevent it, researchers are exploring ways to reduce the “residual” risk, particularly by normalizing elevated levels of lipoprotein(a) or remnant lipoproteins in the blood [3], or by targeting the inflammation process in the vessel wall [4]. It is well known that focal activation of transendothelial transport (TET) of low-density lipoproteins (LDLs) plays a crucial role in atherogenesis. The review analyzes current concepts regarding the mechanisms of LDL TET and its activation in atherogenesis, as well as the prospects for applying these findings in medical practice.

## 2. Activation of Transendothelial Transport of LDLs in Atherogenesis

The cells in the intima and inner third of the media of large arteries receive nutrients, including lipoproteins, not from the *vasa vasorum*, originated from adventitia, but directly from the arterial lumen [5]. The normal intima contains a relatively small number of cells such as fibroblasts, macrophages, lymphocytes, and smooth muscle cells. As a result of the activation of LDL entry into the intima from the blood, which is observed in atherosclerosis [6,7], there is an excess quantity of LDLs in the intima compared to the number of cells that can consume them.

Subsequently, LDL particles become trapped in the intimal layer due to the electrostatic interaction between the positively charged amino acid residues of apolipoprotein B (apoB), the main protein component of LDL, and the negatively charged glycosaminoglycan groups in the extracellular matrix [8]. This process facilitates chemical modifications of apoB, such as oxidation, proteolysis, and glycation. Modified LDLs (mLDLs) are taken up by intimal macrophages via various scavenger receptors, leading to the accumulation of excess cholesterol within the cells, formation of foam cells, and their death [8,9]. The accumulation of LDLs in the intima and the macrophage response to it lead to the development of inflammation, which recruits monocytes and lymphocytes from the blood. The goal of this response is to accelerate the elimination of mLDLs and dead foam cells form the intima. However, continued activation of LDL transport into the intimal layer leads to the accumulation of cholesterol in it, increased inflammation, and, as a result, the formation of an atherosclerotic plaque [10].

There is an opinion that intimal accumulation of LDLs in large arteries is determined not by the rate of LDL TET, but by the particle retention in the intima [8]. According to Schwenke et al. (1989) [11], in rabbits with hypercholesterolemia, there was no increase in LDL TET in pro-atherogenic areas of the arteries, but there was a greater accumulation of LDLs in the intima. In apolipoprotein E-deficient (*Apoe* −/−) mice overexpressing perlecan lacking heparan sulfate, the rate of LDL TET was increased, but LDL retention in the intima was reduced, resulting in less atherosclerosis development [12]. Additionally, the significant role of LDL TET activation in the development of atherosclerosis has also been recognized [7,13,14,15].

**Figure 1:**
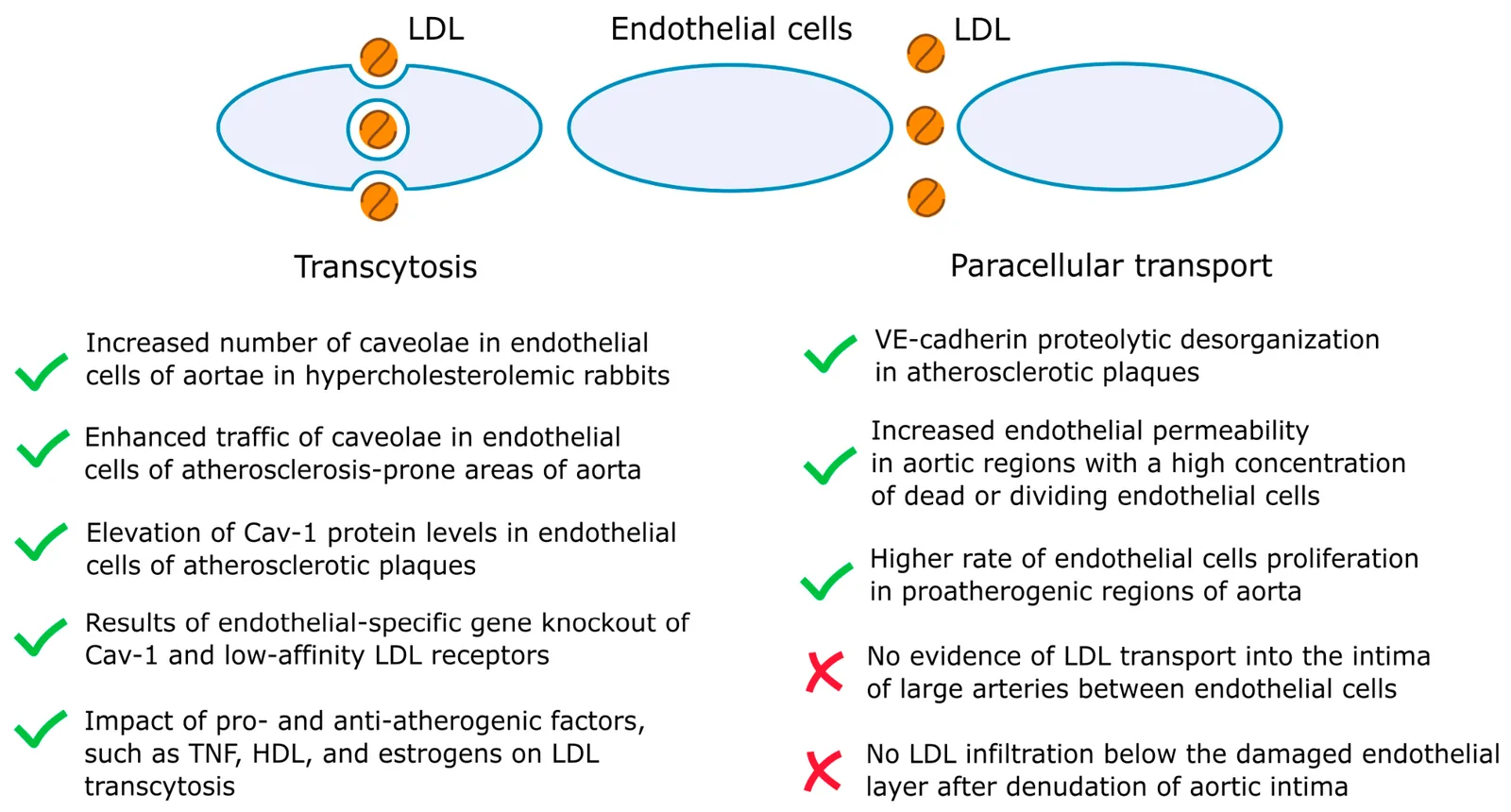
Arguments and counterarguments on the activation of transcytosis and paracellular transport of LDLs through the endothelium in atherosclerosis. Cav-1—caveolin-1; HDL—high-density lipoprotein; LDL—low-density lipoprotein; TNF—tumor necrosis factor. Green checkmark indicates an argument, and red checkmark represents a counterargument. The reference for the first argument supporting the transcytosis mechanism is [6], for the second—[7], for the third—[19], for the fourth—[14,15,20], for the fifth—[17,21,22], for the first argument supporting paracellular transport—[18], for the second—[23,24], and for the third—[25].

The most proven pathway for passage of LDLs and plasma proteins through the endothelium of large arteries is transcytosis [7,16,17]. However, the possibility of paracellular leakage has also been considered [18]. Analysis of the capabilities for both pathways in atherosclerosis is shown in Figure 1.

Proteolytic degradation of the intercellular junction protein VE-cadherin has been detected in the endothelium covering atherosclerotic plaques. Inhibition of this process by the inactivation of m-calpain slowed down the progression of atherosclerosis in LDL receptor deficient (*Ldlr* −/−) mice [18]. Some authors [23,24] point to an increased endothelial permeability in regions of aorta with a high content of dead or dividing endothelial cells (ECs), suggesting the possibility of LDLs passing through opened intercellular junctions. An increase in EC proliferation has been observed in proatherogenic regions of aorta in rabbits with experimental hypercholesterolemia [25]. Nevertheless, none of these studies have directly demonstrated the transport of LDL into intima between endothelial cells. In addition, after the balloon denudation of aortic intima (scraping off the endothelial layer), lipid infiltration of the intima occurred in areas that had undergone re-endothelialization, but not in regions where ECs were still absent [26]. The exact reason why LDLs do not enter the intima through the damaged endothelial layer remains unknown, but it is clear that LDLs pass through endothelium using the transcytosis mechanism.

Activation of LDL vesicular transport through the ECs in atherogenesis is supported by the following observations. Firstly, an increasing number of uncoated vesicles in ECs of aortae in rabbits with induced hypercholesterolemia has been found. Some of these vesicles fuse to form multivesicular daisy-like structures and transcellular channels [6]. Secondly, it was shown that in the ECs of atherosclerosis-prone aortic regions in *Apoe* −/− mice, the proportion of cytoplasmic caveolin vesicles is increased compared to athero-resistant regions. This indicates a more active process of caveolae-mediated transcytosis in these cells [7]. Thirdly, a significant increase in the level of caveolin-1 (Cav-1) was found in atherosclerotic plaques in humans and mice with genetically determined atherosclerosis [19]. EC-specific knockout of the *Cav1* gene in mice delayed the development of atherosclerosis [7,20]. It should be noted that transcytosis of LDLs through ECs is mediated by caveolin-, but not clathrin-coated vesicles [16,20,21,27]. The latter contain specific receptors for LDL (LDLR), fuse with lysosomes, and deliver cholesterol to ECs for their needs [16]. Finally, the LDL transcytosis mechanism is indicated by the involvement of low-affinity LDL receptors in LDL TET activation, as well as by the influence of pro- and anti-atherogenic factors on this process (see Figure 1 and descriptions in Section 3 and Section 4).

## 3. The Involvement of Receptors and Post-Receptor Pathways in LDL Transcytosis Through Endothelial Cells

**Figure 2:**
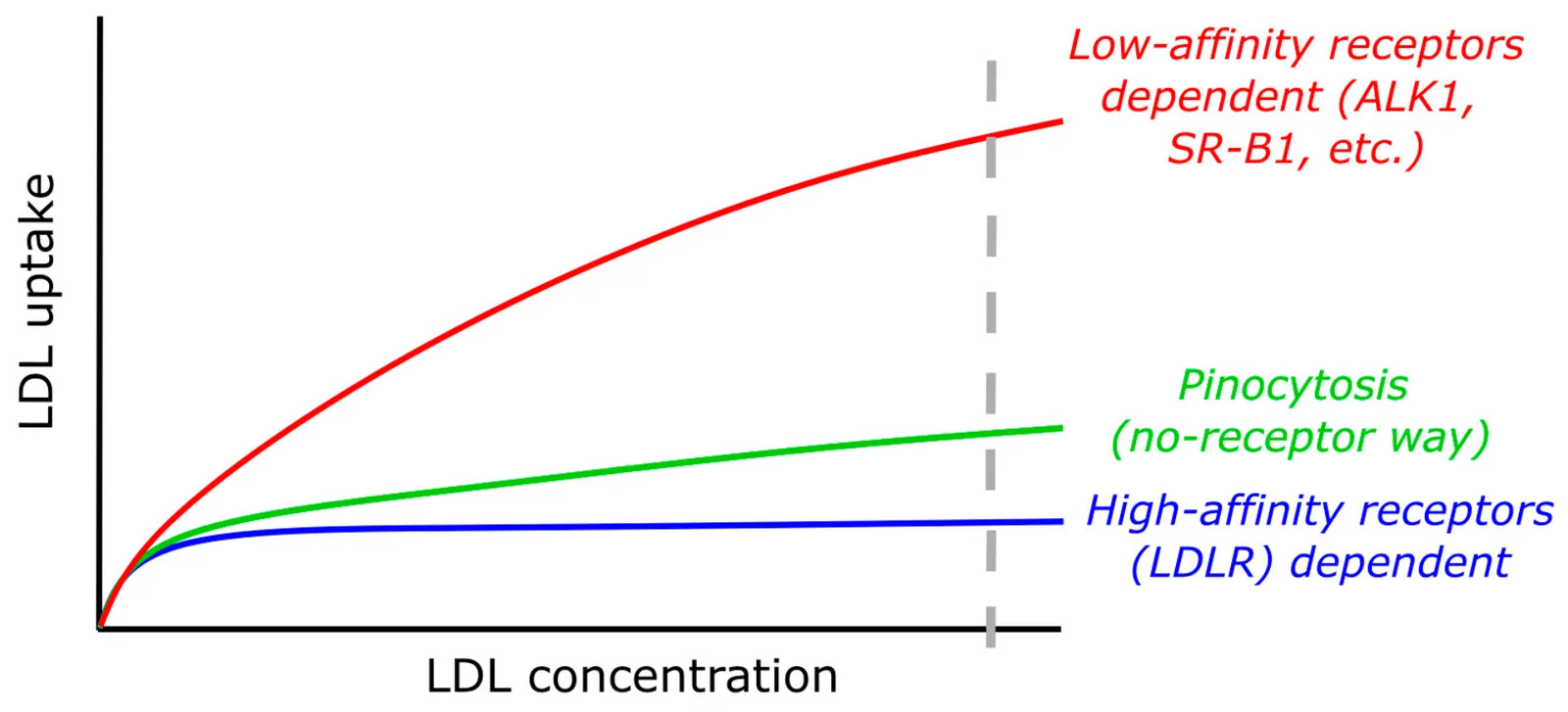
The paths of low-density lipoprotein (LDL) uptake by endothelial cells. The dashed line represents low physiological concentrations of LDLs at 0.5 mg protein/mL. The data for this picture are from [28]. ALK-1—Activin receptor-like kinase-1; LDLR—LDL receptor; SR-B1—scavenger receptor class B type 1.

According to kinetic analysis, even at low concentrations of LDLs (below normal levels of 0.5–1 mg LDL protein/mL), most particles enter ECs through low-affinity receptor-mediated endocytosis, and a much smaller amount through high-affinity LDL receptors or by pinocytosis [28] (Figure 2). In hypercholesterolemia, the proportion of LDLs entering ECs by low-affinity pathways increases. Since high-affinity receptors, such as LDLR, are responsible for delivering cholesterol to cells, their expression is tightly regulated by intracellular cholesterol levels. Therefore, they are not suited for transcellular transport of LDLs. Moreover, it is well known that mutations in the gene of this receptor lead to the development of familial hypercholesterolemia and early atherosclerosis [29].

Recently, two low-affinity receptors for LDLs, SR-B1 (Scavenger Receptor Class B Type 1) and ALK-1 (Activin receptor-like kinase-1), were found in ECs. These receptors are involved in the transport of LDL particles through ECs. Both proteins are found in caveolae and colocalize with LDLs in ECs [13,14,30].

SR-B1 is a multifunctional protein that plays several roles in the body: it is a receptor for high-density lipoproteins (HDLs), a transmembrane transporter for cholesterol and some other lipids, and a scavenger receptor for mLDLs and apoptotic bodies [31]. SR-B1 was also shown to have a low affinity for native LDLs [32]. The specific binding sites on SR-B1 for different types of lipoproteins have not yet been identified, but data from site-directed mutagenesis and *in silico* studies suggest that HDLs, native LDLs, oxidized and acetylated LDLs all bind to the same region of the protein [31]. The latter is a group of three α-helices located in the upper part of the receptor [14,33,34]. Data on the competition between HDLs and LDLs for binding and uptake by ECs suggest a single binding site for SR-B1 with different lipoproteins [17,35].

ALK-1 belongs to the Transforming Growth Factor Beta receptor family, and its canonical ligand is the bone morphogenetic protein type 9 (BMP-9). According to the surface plasmon resonance, the dissociation constant (Kd) for LDL binding to ALK-1 is 200 nM, i.e., ~30 times higher than Kd for LDL binding to LDLR (7 nM). BMP-9 and LDLs do not compete for binding to ALK-1, which indicates that ALK-1 has different binding sites for these ligands [13].

As already mentioned, SR-B1 and ALK-1 are located in caveolae [13,14], and genetic manipulations that lead to an increase or decrease in the expression of genes for these receptors in ECs cause, respectively, up- or down-regulation of LDL TET [13,14,17]. Knockdown of the *Acvrl1* gene, coding ALK-1, caused a decrease in LDL and very low-density lipoprotein (VLDL) TET, but not in HDL or oxidized LDL transport [13]. Deletion of *Scarb1*, coding SR-B1, led to a decline in LDL, oxidized LDL, and VLDL TET [14]. No information has been found regarding the participation of these receptors in the transport of other plasma proteins.

Endothelial cell-specific knockout of *Acvrl1* or *Scarb1*, as well as the administration of blocking antibodies to ALK-1, leads to a slowdown in the development of atherosclerosis in mice with a genetic predisposition to this disorder [14,15]. Thus, these receptors may be responsible for the activation of LDL TET during atherogenesis. The mechanisms triggering LDL transcytosis after activation of these receptors remain poorly understood. It has been established that the interaction of LDLs with SR-B1 leads to the binding of the cytoplasmic region of the receptor to the GDP/GTP exchanger DOCK-4 (Dedicator of cytokinesis 4). The latter activates the GTPase Rac1, facilitating vesicle transcytosis through the effect on the actin cytoskeleton [14,36]. The mechanisms by which ALK-1 receptors initiate transcytosis of LDLs are not well understood, although it has been shown that this process does not depend on the activity of the receptor’s kinase domain, which is responsible for signal transmission from BMP-9 [13].

It is well known that various GTPases of the Rab family regulate the trafficking of intracellular vesicles. Rab5 is localized in early endosomes; Rab7 and 9 in late endosomes; Rab10 in the Golgi apparatus; and Rab4 and 11 in recycling vesicles [37,38]. It is quite possible that a certain set of Rab proteins prevents the fusion of some LDL vesicles with lysosomes and sorts them into transcytotic vesicles [39]. By using gene knockdown approach and immunofluorescence, it was established that Rab4 and 11, which are likely required for the recycling of SR-B1 and ALK-1, as well as Rab10, which is responsible for the transport of LDLs from the trans-Golgi to the basolateral membrane [37], facilitate LDL transcytosis [40]. It is possible that Rab6 is also participating in this process [40]. Interestingly, knockdown of Rab10 or Rab6 led to a reduction in LDL, but not albumin transcytosis [40], which indicates specific pathways for LDL exocytosis. Exocytosis of caveolin-containing vesicles at the basement membrane of ECs is also mediated by SNARE proteins, SNAP, and NSF ATPase [41,42]. The question remains whether such proteins are involved in the regulation of LDL exocytosis.

## 4. Regulation of LDL Transendothelial Transport in Atherosclerosis

**Figure 3:**
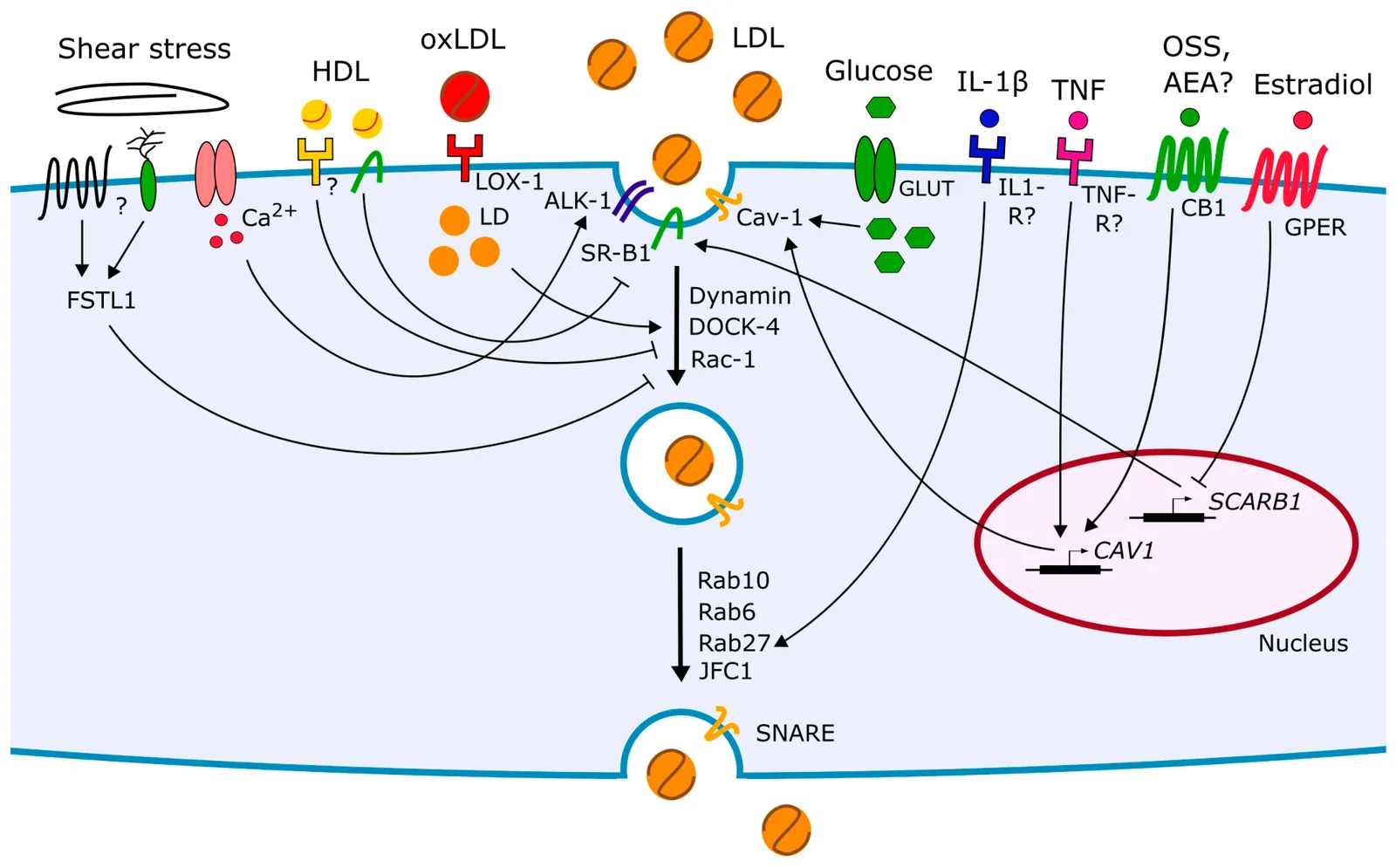
Regulation of low-density lipoproteins’ transcytosis through endothelial cells in atherosclerosis. AEA—N-arachidonoylethanolamine; ALK-1—Activin receptor-like kinase-1; Cav-1—caveolin-1; CB1—cannabinoid receptor 1; DOCK-4—Dedicator of cytokinesis 4; FSTL1—follistatin-like protein-1; GLUT—glucose transporter; GPER—G-protein-coupled estrogen receptor; HDL—high-density lipoproteins; IL-1β—interleukin-1β; JFC1—synaptotagmin-like protein 1; LD—lipid droplets; LDL—low-density lipoproteins; LOX-1—oxidized LDL receptor-1; OSS—oscillatory shear stress; oxLDL—oxidized LDL; R—receptor; SNARE—soluble NSF attachment protein receptors; SR-B1—scavenger receptor class B type 1; TNF—tumor necrosis factor; ?—unknown or hypothetical receptors/ligands participating. References to the sources of information used for this picture are provided in Section 4. Arrows represent activation signals, blunt arrows indicate inhibitory signals, vertical bold arrows denote the direction of vesicle traffic.

A number of factors potentially contribute to the activation of LDL TET during atherogenesis (Figure 3).

### 4.1. Shear Stress

Hemodynamic forces, such as blood flow turbulence, shear stress, and pulsatile stretching of the vascular wall, are the main factors that can cause focal activation of LDL TET in atherosclerosis. Indeed, it has been shown on perfused aorta and isolated ECs that short-term (several hours) but not long-term (24 h) shear stress enhances the passage of LDLs through the endothelial cell layer [43,44]. It is quite likely that mechanoreceptors responsible for the activation of ECs do not respond to unidirectional flow of a single speed and force, but to a change in flow direction, which creates “oscillatory” stress. The influence of oscillatory stress on LDL TET is evidenced by the predominant localization of atherosclerotic lesions in arterial regions affected by turbulent blood flow, as well as by the increased expression of *Cav1* and *Scarb1* in these regions [7,45].

Under oscillatory stress, the expression of the endocannabinoid receptor gene, *CB1*, in ECs is increased [46]. As shown in a number of studies, this receptor is involved in the development of atherosclerosis and metabolic syndrome [47]. Activation of this receptor leads to an increase in LDL TET via Gi-protein/c-Jun/Cav-1 signaling [46]. The mechanisms of CB1 activation under oscillatory stress, the involvement of the endogenous receptor’s lipid ligands, such as N-arachidonoylethanolamine (AEA), in this process, and the possible role of CB1 as a mechanoreceptor require clarification.

Turbulent flow activates a number of mechanosensors in ECs, such as Piezo1, polycystins, and connexin-37, also functioning as Ca^2+^ channels [48,49,50]. In turn, the influx of Ca^2+^ into the cytosol can activate the internalization of various molecules/particles, including LDLs, via Ca^2+^-calmodulin-mediated activation of the cdc42 GTPase, which leads to changes in actin polymerization [51,52].

One of the main roles in mechanosensing is attributed to glycocalyx [53,54]. Thinning of the glycocalyx layer due to a decrease in the amount of hyaluronate and heparan sulfates has been observed in areas of blood vessels predisposed to atherosclerosis [55]. A reduction in the thickness of the glycocalyx, with signs of its defragmentation, has been detected in human atherosclerotic plaques [56]. Laminar shear stress increases, while turbulent flow decreases the expression of glycocalyx proteins on the surface of ECs [57]. The latter process occurs, in particular, because of secretion of the active form of the matrix metalloproteinase-9 due to pro-inflammatory activation of ECs caused by oscillatory flow [58,59].

It is essential to determine the impact of glycocalyx proteins and their carbohydrate components on LDL TET, as well as to explore which signaling events triggered by specific mechanoreceptors are responsible for their influence on the transport. Moreover, the role of a number of signaling pathways that respond to hemodynamic stress, such as Klf-2/4, Yap/Taz, and Wnt/β-catenin, in LDL TET remains unclear [60,61,62].

According to *in vitro* data, the conditioned medium obtained from endothelial cells cultured under laminar but not oscillatory shear stress exposure suppressed transcytosis of protein particles with a similar size to LDLs and had an anti-inflammatory effect on ECs. Further proteome and functional analysis revealed that follicistatin-like protein-1 (FSTL1) exerted such an effect in the conditioned medium; its level increased under laminar stress. The suppressive effect of FSTL1 on LDL TET is likely related to a decrease in inflammasome activity and BMP-4 production [63].

### 4.2. High-Density Lipoproteins

Transendothelial transport of LDLs can be affected by HDLs [64], as well as by their main apolipoprotein, apolipoprotein A-1, possibly due to a competition for binding to the SR-B1 receptors [17,35]. However, whether it is also a result of signaling effects of HDLs remains understudied. In this regard, it is worth noting that the lipid-derived component associated with apolipoprotein M in HDLs, sphingosine-1-phosphate (S1P), may play a role in enhancing the barrier properties of endothelial cells. This occurs through the interaction of S1P with the S1P receptor (S1PR1) on the surface of ECs. Subsequently, the activation of kinases PI3K and Akt leads to an increase in the activity of GTPase Rac1 and endothelial NO synthase (eNOS), which ultimately causes changes in the actin cytoskeleton, stabilizing intercellular contacts [65]. The elevation of plasma concentration of S1P (including the HDL-associated one) led to a decrease in the permeability of the vascular wall to LDL. This was accompanied by a slowing down of the development of atherosclerosis in mice with atherogenic genetic backgrounds [66].

### 4.3. Hypercholesterolemia

Another factor influencing the permeability of ECs of large arteries into LDL is hypercholesterolemia. As mentioned earlier, in the ECs of rabbit aortas with experimental hypercholesterolemia, there is an increase in the number of caveolae and the formation of rosette-like structures and transcellular channels, which are involved in the process of LDL transcytosis [6]. Conversely, in *Ldlr* knockout mice that were treated with anti-ApoB antisense oligonucleotides, a clear decrease in LDL cholesterol levels in the blood plasma was observed, along with a reduction in aortic permeability to LDLs [67]. A 5-day incubation of ECs with cholesterol-rich β-VLDLs results in the intracellular accumulation of cholesterol and an increase in the transcellular transport of LDLs and albumin [68]. It should also be noted that in mice with overexpression of the *PCSK9* gene (and, consequently, with a significant increase in plasma LDL levels), the atheromatous aorta contains approximately 30% ECs with perilipin-2-rich lipid droplets [69]. For some reason, after LDLs are taken up by ECs, some of the particles undergo degradation within lysosomes. This could be due to the scavenger capture of mLDLs (by the oxidized LDL receptor-1 (LOX-1), expressed in ECs [70]), the level of which increases in proportion to the degree of hypercholesterolemia. Cholesterol is then deposited in lipid vacuoles as cholesterol esters. Another possible component of lipid vacuoles in ECs may be triglycerides [71,72].

Perhaps the activation of LDL TET in hypercholesterolemia is related to the accumulation of lipid vacuoles in endothelial cells. Deposition of lipid droplets in ECs due to the EC-specific knockout of the adipose triglyceride lipase gene, leads to a decrease in eNOS gene expression, accompanied by a reduction in NO production, and to an activation of inflammatory processes in ECs, likely due to endoplasmatic reticulum (ER) stress [71,72]. In addition to the action of neutral lipase, lipid vacuoles in ECs can also be destroyed through auto-(lipo)phagy [69,73]. Consequently, in those ECs of the atherosclerotic plaque where autophagy was decreased, the lipid content was higher [69]. In turn, impaired autophagy in ECs, particularly when induced by the addition of oxidized LDL, led to increased transcytosis of LDLs [73]. Stimulating lipophagy in ECs can be considered as a measure to fight atherosclerosis [69], by reducing the entry of LDL particles into the vascular wall. Another way to remove lipid vacuoles from ECs is through the formation of extracellular vesicles [74].

### 4.4. Hyperglycemia

One more factor that could affect LDL TET is diabetes mellitus. In patients with this disorder, an increase in the rate of LDL elimination from the bloodstream is observed, which likely occurs due to an acceleration of the passage of these particles through the endothelium [75]. *In vitro* studies have shown that high glucose concentrations increase the LDL TET by the elevating content of Cav-1 in cells due to the inhibition of its degradation [76]. In a yet-to-be-fully-understood mechanism, glucose enters the cell and slows down the nuclear-cytoplasmic translocation of the deacetylase Sirt6, which, through deacetylation of Cav-1, directs the protein to lysosomes [77]. On the other hand, in diabetes mellitus, the synthesis of Cav-1 in EC increases due to the stimulating effect of advanced glycation end products on RAGE (receptor for advanced glycated end products)—NF-κB (nuclear factor-κB) signaling [78]. The promoter region of the *CAV1* gene contains an element activated by NF-kB [21,79].

### 4.5. Cytokines

Proinflammatory cytokines, such as TNF (tumor necrosis factor) and IL (interleukin)-1β, which are elevated in obesity and metabolic syndrome and actively produced in atherosclerotic plaques, stimulate LDL TET by the NF-κB pathway. This activation leads to an up-regulation of expression of genes, coding caveolae proteins Cav-1 and cavin-1 [21,80]. According to other studies, the effect of these cytokines on LDL TET was not accompanied by changes in the expression of the *CAV1* gene [81,82], and IL-1β did not affect the content of SR-B1 and ALK-1 transporters in ECs [82]. Interestingly, IL-1β increases the LDLR protein level in the EC, and it is through this transporter that IL-1β stimulates LDL TET [82]. It is unclear how IL-1β leads to an increase in LDLR content in ECs, given that both gene expression and stability of the LDLR protein are negatively regulated by the level of cholesterol in the cell; it is expected that under conditions of hypercholesterolemia, the level of LDLR in cells should be decreased [83]. Another possible mechanism of the action of IL-1β is the activation of Rab27a and its effector JFC1 (synaptotagmin-like protein 1), which are involved in exocytosis near the basement membrane [82].

Unlike TNF and IL-1β, the cytokines interferon-γ, IL-6 and IL-4, as well as the anti-inflammatory adipokine adiponectin, did not affect LDL TET [81,82,84]. At the same time, IL-6 stimulated the transport of HDLs through ECs by enhancing the synthesis of endothelial lipase, the HDL-binding protein [85]. In contrast, acute phase proteins, such as C-reactive protein and serum amyloid A, increased LDL TET [80,86,87]. The mast cell mediator, histamine, suppressed the TET of LDLs and albumin. This effect was only observed after 24 h of incubation, and not at earlier time points. The suppression was mediated by H1-histamine receptors, but not H2-receptors [88].

### 4.6. Estrogens

Finally, it has been established that estrogens, hormones that reduce the atherogenicity of the lipoprotein spectrum in women [89], also have an effect on LDL TET [22]. The addition of estradiol to coronary artery ECs caused a decrease in LDL transcytosis. This effect was mediated by the interaction of estrogens with membrane receptors (GPER, G-protein-coupled estrogen receptors), the activation of which causes a decrease in *SCARB1* expression. It has been shown that the expression *SCARB1* in endothelial cells of male coronary arteries is higher than in the same cells from female coronary arteries [22].

### 4.7. Multiple Risk Factors

The studies cited above describe the effects of some pro- and anti-atherogenic factors separately. However, since a person usually has several risk factors, it would be valuable to assess the impact of various combinations of these factors on LDL TET. This question remains unanswered.

## 5. Promising Targets for Normalizing the Transport of LDLs Through the Endothelial Barrier

**Figure 4:**
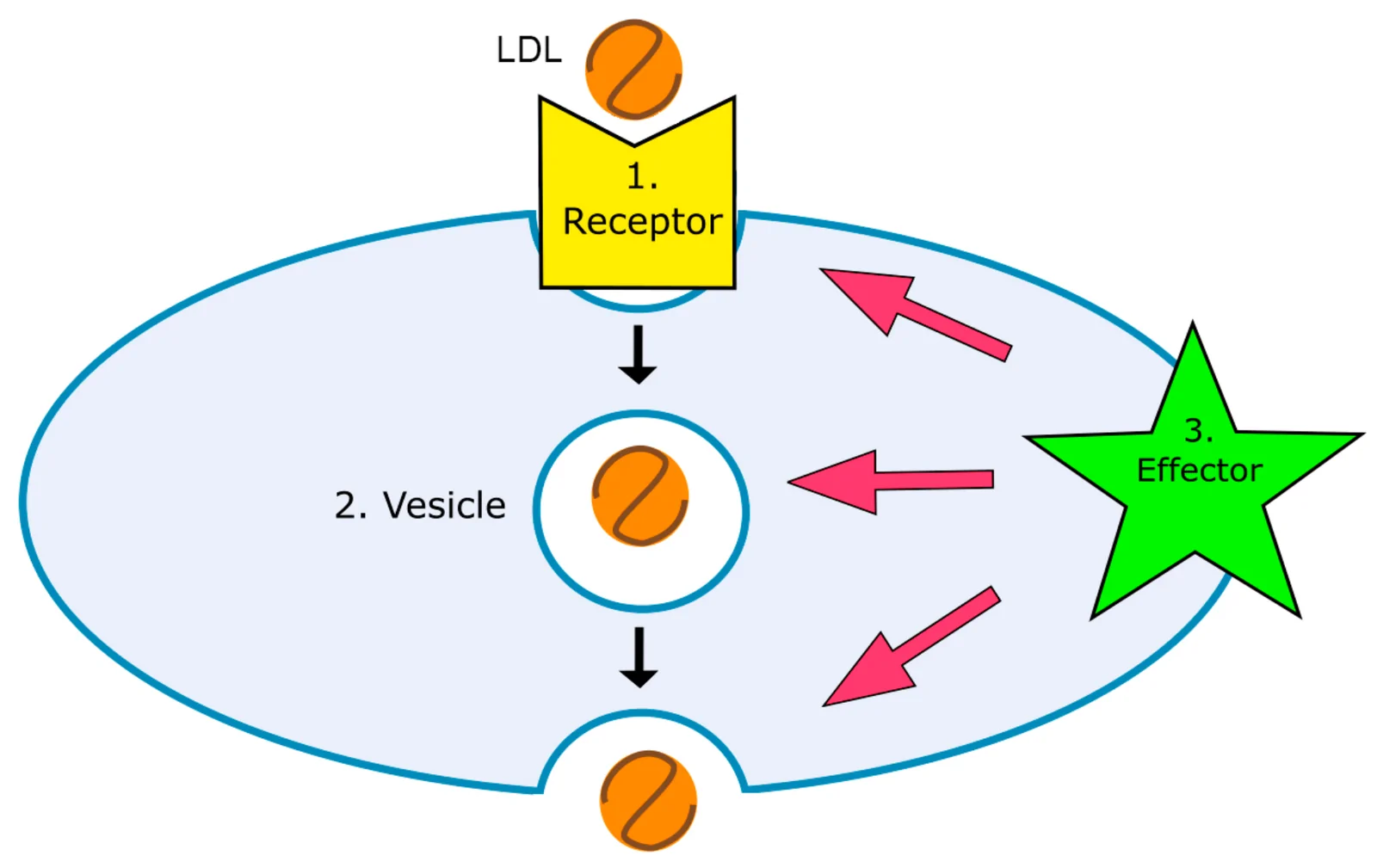
The types of molecular targets for reducing LDL endothelial transcytosis in atherosclerosis: low-affinity LDL receptors (1), transcytotic vesicle machinery (2), and effectors of LDL transcytosis (3).

There are three approaches to targeting activated LDL transcytosis in atherosclerosis, by affecting: (1) low-affinity LDL receptors, (2) vesicular pathway, and (3) the effectors of LDL transport (Figure 4). The specific targets and potential drugs for them are listed in Table 1.

The most obvious approach would be to block the receptors involved in LDL transcytosis—ALK-1 and/or SR-B1. Moreover, inhibiting ALK-1 appears to be more promising, since this receptor is localized only in ECs [15], while SR-B1 is also found in macrophages [96]. Moreover, the latter is also a receptor for HDLs and provides a number of antiatherogenic functions for both macrophages and ECs [97,98]. Blocking monoclonal antibodies to ALK-1 have been developed that reduce LDL TET but do not affect ALK-1 signaling activated by BMP-9 [15]. In turn, adding BMP-9 to ECs caused a decrease in the amount of ALK-1 on the plasma membrane and, as a result, LDL internalization [91]. It is likely that analogues of BMP-9 can be developed that do not activate BMP signaling but still reduce LDL internalization. However, ALK-1 and SR-B1 have relatively low affinity for LDLs [13,14] and, apparently, low specificity, which is especially characteristic of SR-B1 [14,33]. Moreover, both receptors perform LDL transport along with other functions, and the former one may not be their main function. The low specificity of LDL transport receptors is evidenced, in particular, by observations of the identical rate of penetration of modified and unmodified LDLs into the aorta of rabbits [99], as well as by the increased transport of albumin into proatherogenic areas of the aorta in rabbits [100]. It is quite possible that a far-from-complete spectrum of LDL transport receptors has been identified. Accordingly, if only one of the receptors is blocked, LDL transport through cells will be carried out via other receptors. This means that one should not expect strong effects from the inhibition of certain LDL receptors. Nevertheless, administering anti-ALK-1 monoclonal antibodies to mice with *Ldlr* knockout led to a 50% reduction in the area of atherosclerotic lesions, and the apoB content in the intima decreased by 60% [15]. The results of further studies of these antibodies will help clarify the issue of the effectiveness of this approach. It is also promising to investigate the possibility of using peptide fragments of apoB or its analogues to block TET LDL [90].

Another approach to influencing the LDL transport mechanism is to block the formation and trafficking of intracellular vesicles. This can be done, for example, by using Cav-1 inhibitors. Well-known candidates for this are statins. By inhibiting cholesterol synthesis, they cause a depletion of cholesterol in cell membrane lipid rafts. This leads to a reduction in the localization of Cav-1 on the membrane of ECs and, consequently, an increase in the activity of eNOS [92,93]. However, it is not clear whether statins affect LDL transcytosis [101]. The microtubule inhibitor colchicine is approved for the treatment of atherosclerosis, primarily as an anti-inflammatory agent that disrupts inflammasome assembly [94]. Potentially, it can also be used as an inhibitor of vesicular transport [95]. Other possible targets of the intervention may include the protein dynamin, which is responsible for the formation of endocytic vesicles [36], transport GTPases, particularly those involved in exocytosis—Rab10 and Rab27a [40,82]—as well as SNARE proteins, which ensure the mechanism of fusion of exocytic vesicles with the basement membrane [36]. However, a significant limitation of this approach is the lack of specificity in blocking transport of LDLs.

In addition to the approaches mentioned above, it is also possible to try to influence the activity of certain effectors and signaling pathways that regulate LDL TET, such as mechanoreceptors, endocannabinoid receptors, and FSTL1/BMP-4, and by increasing the concentration of HDLs or its individual components, such as S1P. Additionally, inhibiting RAGE and scavenger receptors (SRA, LOX-1, etc.) on ECs may also be effective in regulating LDL TET.

Finally, it is critically important to develop methods for delivering drugs into the atherosclerotic plaque. Targetness can be achieved, for example, through inflammatory molecules synthesized in the atherosclerotic plaque. Delivery of NLPR3 inflammasome siRNA in cationic liposomes containing a peptide ligand for vascular cell adhesion protein-1 to atherosclerotic lesions in *ApoE*-deficient mice resulted in a reduction in LDL entry into the aortic intima and suppression of atherosclerosis development [102]. Identifying new molecules in ECs involved in LDL TET in atherosclerosis will also contribute to the development of targeted therapy for this disease.

## 6. Conclusions

The main mechanism of LDL transport through the endothelium of large arteries is caveolae-mediated transcytosis. This process begins with the interaction of LDLs with low-affinity receptors, which include, at least, ALK-1 and SR-B1. The mechanisms that prevent the fusion of endocytic vesicles in ECs with lysosomes and direct the former along the transcytosis pathway need to be investigated. At the same time, the involvement of several Rab proteins that facilitate the transfer of vesicles containing LDLs to the basement membrane has been described. A number of factors involved in atherogenesis regulates the intensity of LDL passage through ECs: shear stress, HDLs, hypercholesterolemia, hyperglycemia, estrogens, and some cytokines. All the mentioned participants in LDL transcytosis—receptors, vesicle proteins, Rabs, as well as the LDL TET effectors and their receptors and intracellular signaling pathways—can be potential targets for anti-atherosclerotic therapy. At the same time, targeted delivery of LDL transcytosis inhibitors into the atherosclerotic plaque is crucial, and this area also needs to be elaborated.

## Figures and Tables

**Table 1:** Potential molecular targets and drugs for normalizing LDL transendothelial transport in atherosclerosis.

Target	Potential Drugs	Experimental Evidence	Ref.
Receptors for LDLs			
ALK-1	Anti-ALK-1 antibodies	Inhibition of atherosclerosis development on *Ldlr* −/− mice	[13]
	APOB12	Reducing LDL uptake by ECs	[90]
	BMP-9 and its analogues	Induction of ALK-1 internalization into ECs	[91]
SR-B1	Anti-SR-B1 antibodies	Lowering LDL incorporation into aorta in experiments with mice	[14]
	SR-B1 inhibitors (BLT-1)	Attenuation of LDL uptake by ECs	[14]
	APOB18	Reducing atherosclerosis in *Ldlr* −/− mice	[90]
Vesicles formation and traffic			
Cav-1	Statins	Reduction in Cav-1 membrane localization in EC	[92,93]
Microtubules	Colchicine	Inhibition of vesicular traffic in hepatocytes, reduction of atherosclerosis progression in humans	[94,95]
Dynamin	Dynasore	Decreasing albumin transcytosis through ECs	[36]
DOCK-4	DOCK-4 inhibitors	Suppression of SR-B1-dependent LDL uptake and transcytosis in ECs	[14]
Rac1	Rac1 inhibitors	Suppression of LDL uptake by ECs	[14]
Rab10	Rab10 inhibitors	Suppression of LDL transcytosis in ECs	[40]
Rab27a	Rab27a inhibitors	Inhibition of IL-1β-induced LDL accumulation in aorta of *Ldlr* −/− mice	[82]
JFC1	JFC1 inhibitors	Inhibition of IL-1β-induced LDL transcytosis in ECs	[82]
SNARE	SNARE inhibitors	Inhibition of albumin internalization by ECs	[36]
LDL transytosis effectors			
HDL receptors	Agonists for HDL receptors	HDLs inhibit LDL transcytosis in ECs	[17,35]
FSTL1	FSTL1 or its analogues	Suppression of endothelial transcytosis of protein particles with a similar size to LDLs	[63]
CB1	CB1 inhibitors	Attenuation of atherosclerosis in *Ldlr* −/− mice	[46]

ALK-1—Activin receptor-like kinase-1; APOB12 and APOB18—N-terminal fragments of apolipoprotein B protein accounting for 12% and 18%, respectively, of its total sequence.; BLT-1—block lipid transport-1; Cav-1—caveolin-1; CB1—cannabinoid receptor 1; DOCK-4—Dedicator of cytokinesis 4; EC—endothelial cell; FSTL1—follistatin-like protein-1; HDL—high-density lipoprotein; IL-1β—interleukin-1β; JFC1—synaptotagmin-like protein 1; LDL—low-density lipoprotein; *Ldlr*—gene, coding for LDL receptor; SNARE—soluble NSF attachment protein receptors; SR-B1—scavenger receptor class B type 1.

## Data Availability

No new data were created or analyzed in this study. Data sharing is not applicable to this article.

## Abbreviations

The following abbreviations are used in this manuscript:

LDL	low-density lipoprotein
TET	transendothelial transport
apoB	apolipoprotein B
mLDL	modified LDL
EC	endothelial cells
Cav-1	caveolin-1
LDLR	LDL receptors
SR-B1	Scavenger Receptor Class B Type 1
ALK-1	Activin receptor-like kinase-1
HDL	high-density lipoprotein
BMP-9	bone morphogenetic protein type 9
VLDL	very low-density lipoprotein
DOCK-4	Dedicator of cytokinesis 4
FSTL1	follicistatin-like protein-1
S1P	sphingosine-1-phosphate
eNOS	endothelial NO synthase
NF-κB	nuclear factor-κB
TNF	tumor necrosis factor
IL	interleukin
